# Affimer reagents enable targeted delivery of therapeutic agents and RNA via virus-like particles

**DOI:** 10.1016/j.isci.2024.110461

**Published:** 2024-07-05

**Authors:** Sophie E. Roberts, Heather L. Martin, Danah Al-Qallaf, Anna A. Tang, Christian Tiede, Thembaninkosi G. Gaule, Albor Dobon-Alonso, Ross Overman, Sachin Shah, Hadrien Peyret, Keith Saunders, Robin Bon, Iain W. Manfield, Sandra M. Bell, George P. Lomonossoff, Valerie Speirs, Darren C. Tomlinson

**Affiliations:** 1 School of Molecular and Cellular Biology, University of Leeds, Leeds, UK; 2 Astbury Centre for Structural and Molecular Biology, University of Leeds, Leeds, UK; 3 Institutue of Cardiovascular & Metabolic Medicine, University of Leeds, Leeds, UK; 4 Leaf Expression Systems, Norwich Research Park, Norwich, UK; 5 Department of Biochemistry and Metabolism, John Innes Centre, Norwich Research Park, Norwich, UK; 6 Leeds Institute of Cardiovascular and Metabolic Medicine, School of Medicine, University of Leeds, Leeds, UK; 7 Leeds Institute of Medical Research at St James’s, St James’s University Hospital, University of Leeds, Leeds, UK; 8 Institute of Medical Sciences, University of Aberdeen, Aberdeen, UK

**Keywords:** Virology, Molecular biology

## Abstract

Monoclonal antibodies have revolutionized therapies, but non-immunoglobulin scaffolds are becoming compelling alternatives owing to their adaptability. Their ability to be labeled with imaging or cytotoxic compounds and to create multimeric proteins is an attractive strategy for therapeutics. Focusing on HER2, a frequently overexpressed receptor in breast cancer, this study addresses some limitations of conventional targeting moieties by harnessing the potential of these scaffolds. HER2-binding Affimers were isolated and characterized, demonstrating potency as binding reagents and efficient internalization by HER2-overexpressing cells. Affimers conjugated with cytotoxic agent achieved dose-dependent reductions in cell viability within HER2-overexpressing cell lines. Bispecific Affimers, targeting HER2 and virus-like particles, facilitated efficient internalization of virus-like particles carrying enhanced green fluorescent protein (eGFP)-encoding RNA, leading to protein expression. Anti-HER2 affibody or designed ankyrin repeat protein (DARPin) fusion constructs with the anti-VLP Affimer further underscore the adaptability of this approach. This study demonstrates the versatility of scaffolds for precise delivery of cargos into cells, advancing biotechnology and therapeutic research.

## Highlights

• HER2-binding Affimers identified that are internalized by HER2-overexpressing cells
• Internalization is specific and delivers cytotoxic and virus-like particle payloads
• Virus-like particle cargo is effectively expressed intracellularly
• Expression of virus-like particle cargo is Affimer dose-dependent

## Graphical abstract

**Figure:**
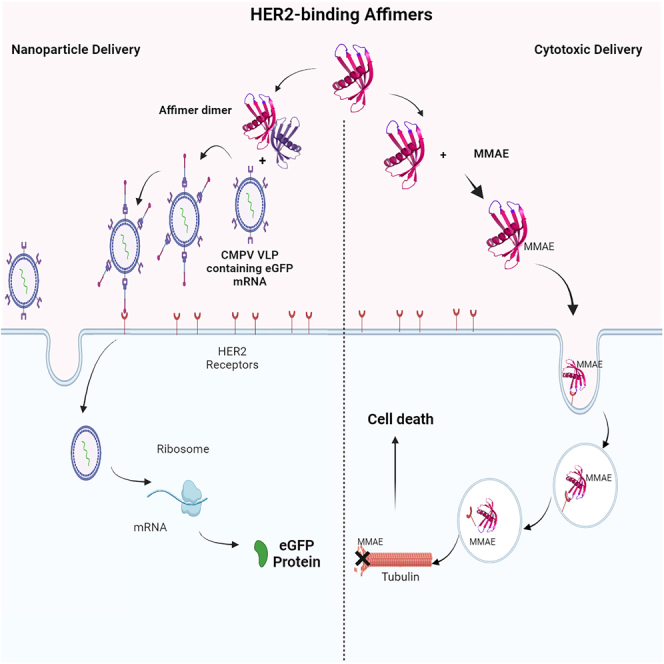

## Introduction

Cancer is a leading cause of death worldwide^1^ and highly heterogeneous, creating scope and need for stratified medicine approaches. Breast cancer is a clear example of this, with the presence or absence of estrogen and HER2 receptors defining treatment strategy, the former being tamoxifen-responsive and the latter being trastuzumab ± pertuzumab-responsive.^2^^,^^3^^,^^4^^,^^5^ This demonstrates the requirement and effectiveness of therapeutics that target specific cell populations. To date, the majority of such targeted therapies have been provided by monoclonal antibodies (including a small group of antibody-cytotoxic drug conjugates), targeting cell surface receptors overexpressed on cancer cells compared to their non-cancerous neighbors.^6^ However, it has become clear that there is scope for smaller and more easily produced proteins to achieve similar impacts. Indeed, in recent years, a number of non-immunoglobulin scaffolds (or scaffold-based binding proteins [SBPs]) have been developed that overcome some of the limitations associated with antibodies, notably their large size, which decreases their ability to penetrate solid tumors, slow blood clearance, and expense to produce. SBPs have many advantageous features, such as small size, high solubility, high stability, and lack of disulfide bonds, and include, among others, designed ankyrin repeat proteins (DARPins) and Affibodies as well as the Affimers^7^^,^^8^^,^^9^ utilized in this study. These easily producible proteins have been used for a range of biological and therapeutic applications to date.^10^ Indeed, HER2 has been targeted with SBP-conjugated cytotoxics, notably an Affibody-monomethyl auristatin E (MMAE) conjugate with picomolar affinity^11^^,^^12^ and nanomolar IC_50_ in HER-2-expressing SK-BR-3 cells^13^ that is comparable to the HER2-targeting monoclonal antibody-conjugate trastuzumab emtansine.^14^ This demonstrates the applicability of SBP conjugates toward this target for cancer therapy approaches.

However, such targeted conjugates, antibody and SBP, still face issues from biodistribution and pharmacokinetics,^15^ which can lead to side effects including cardiotoxicity and the development of drug resistance.^16^ One methodology to overcome these issues is nanoparticle encapsulation. There exist many types of synthetic nanoparticle; however, only a few have made it to the clinic due to discrepancies between *in vitro* characteristics and subsequent *in vivo* behavior as well as complex regulatory requirements.^17^ Attention has, therefore, turned to nature’s ready-made delivery system in the form of viruses, from which virus-like particles (VLPs) have been derived.^18^ VLPs are non-infectious, self-assembling viral capsids that can encapsulate cargos ranging from cytotoxics to proteins.^16^ A range of expression platforms have been used for producing VLPs, including bacteria, plants, and mammalian cells.^18^ Indeed HER2-targeting VLPs have been used successfully as vaccine therapies in pre-clinical studies, using a variety of VLP platforms.^8^^,^^19^ All these studies required modulation of the VLP capsid to permit targeting to the HER2 expressing cells; such modification can be expensive and problematic.^16^ Here, we show that HER2-binding Affimers used in dimeric format with our previously reported CPMV VLP-binding Affimer^19^ can efficiently deliver plant-derived VLPs with RNA payloads into HER2-positive cell lines. As both SBPs and CPMV VLPs can be readily produced at low cost and in high yield,^8^^,^^19^ these results highlight the potential use of SBP-VLP reagents as a cost-effective biotherapeutic approach that could be used for numerous indications, such as cancer therapeutics and vaccine delivery.

## Results

### Identification of Affimers that bind cellularly expressed HER2

**Figure 1:**
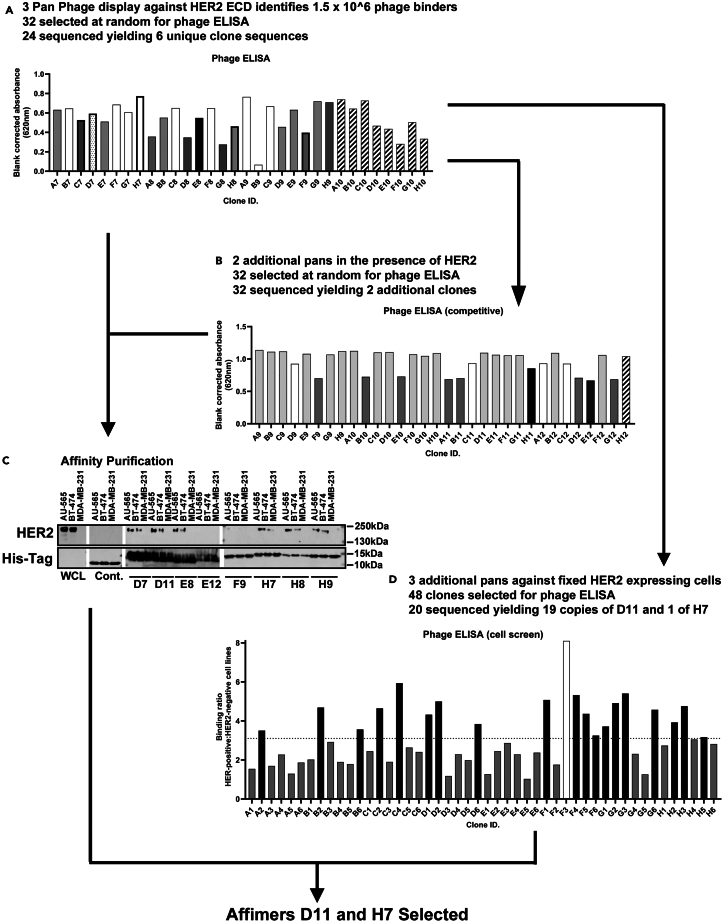
Identification of Affimers that bind cellularly expressed HER2 (A) Phage ELISA of 32 Affimer clones identified as binding the ECD of HER2. Identical clones are shown with the same shading. (B) Phage ELISA of 32 Affimer clones identified as binding the ECD of HER2 in the presence of HER2. Identical clones are shown with the same shading. (C) Affinity precipitation of endogenous HER2 from HER2-overexpressing cell lines (AU-565 and BT-474) and the HER2-negative cell line (MDA-MB-231) using HER2-binding Affimers identified by phage screening against the ECD of HER2. (*n* = 3 independent experiments with representative blots shown; white lines delineate individual membranes; all blots were run concurrently). (D) Phage ELISA of 48 Affimer-clones identified as preferential binding fixed HER2-expressing cells (MDA-MB-453) compared to HER2-negative cells (MCF7). An arbitrary cutoff of 3 was used to determine which clones would be taken forward for DNA sequence analysis identifying the two unique binding Affimers D11 and H7. Identical clones are shown with the same shading. ECD, extracellular domain (amino acids 1-652); WCL, whole-cell lysates; Cont., control Affimer (Variable regions AAAA; AAE).

Initially eight Affimers that bind to the extracellular domain (ECD) of HER2 (amino acids 1–652) were isolated using phage display (Figures 1A and 1B).^7^^,^^8^ The ability of these Affimers to bind native HER2 expressed by breast cancer cells, as opposed to the Fc-conjugated version used in phage screening, was assessed by affinity precipitation. Six Affimers—H7, H8, H9, D7, D11, and E8—precipitated HER2 from lysates of HER2-overexpressing cell lines, AU-565 and BT-474, but not from the HER2-negative cell line MDA-MB-231 (Figure 1C). This demonstrates the ability of HER2-binding Affimers to recognize HER2-expressing breast cancer cells. In parallel, an attempt to isolate Affimers with high affinity for cellularly expressed HER2 was undertaken. To this end we screened the enriched phage pool from the protein screen against monolayers of fixed cancer cells expressing moderate levels of HER2 (MDA-MB-453; Figure S1) with a HER2-negative cell line (MCF-7) as negative selection pan. From 20 clones sequenced following phage ELISA (Figure 1D), two Affimers (D11 and H7) were isolated that had been previously identified by our standard approach (Figures 1A and 1B). These two Affimers were then taken forward for subsequent characterization, as they showed the most promise for successful binding to HER2 on live cells.

### HER2-binding Affimers are internalized by breast cancer cell lines expressing native HER2

**Figure 2:**
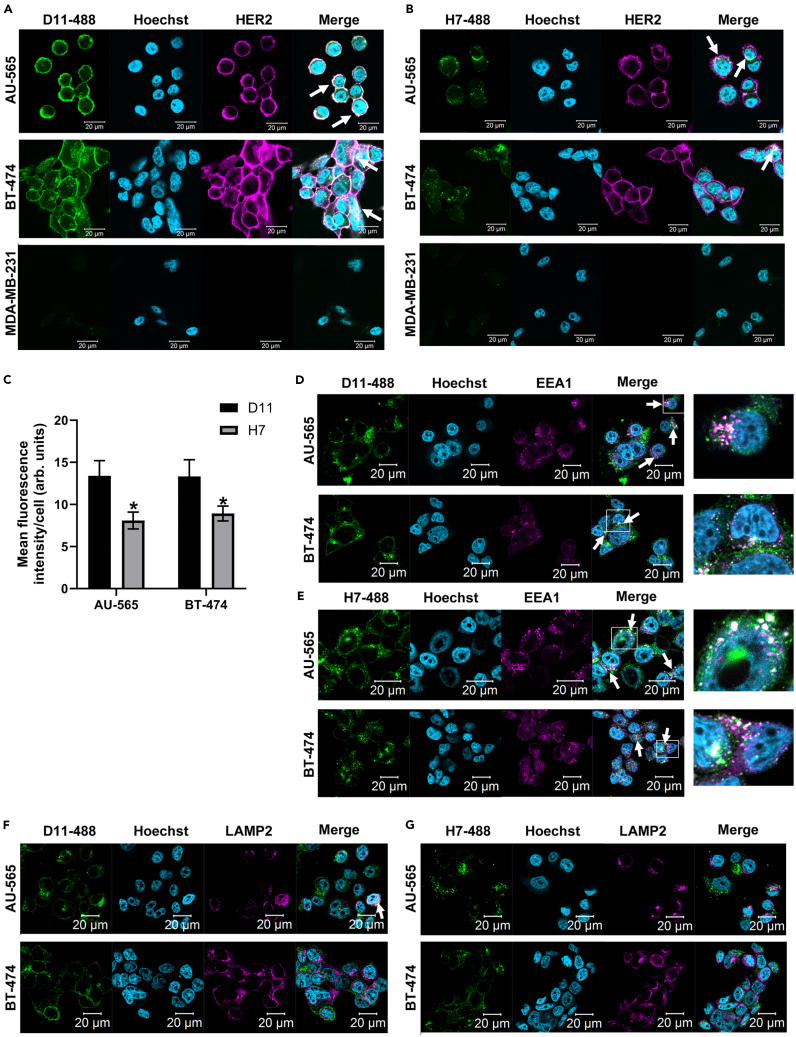
Affimers D11 and H7 colocalize with HER2 and show internalization into HER2-positive cells AlexaFluor 488-conjugated Affimers (green) D11 (A) and H7 (B) were incubated with HER2-positive (AU-565 and BT-474) and -negative (MDA-MB-231) cell lines for 1 h before fixation. Cells were stained for HER2 (pink) and with Hoechst (blue). The internalization of the Affimers D11 and H7 was assessed as mean AlexaFluor 488 intensity per cell (C). The mechanism of internalization was assessed with costaining for EEA1 [pink, (D) and (E)] and LAMP2 [pink, (F) and (G)]. Colocalization of Affimers with HER2/EEA1/LAMP2 is shown by arrows. Images were taken on a Zeiss confocal microscope with a 40x magnification under the same exposure for AlexaFluor 488-conjugated Affimers. Images were analyzed using ZenLite and ImageJ software. Data are mean ± SEM, *n* = 3 independent experiments, with representative images shown, unpaired t test. ∗*p* < 0.05 (C).

To be of use as a delivery mechanism, it is necessary for the HER2-binding Affimers to be selectively internalized by HER2-positive breast cancer cell lines, as seen with other SBPs^11^^,^^13^^,^^14^^,^^20^^,^^21^ and antibody-drug conjugates such as trastuzumab emtansine.^22^ To visualize internalization, AlexaFluor 488 was conjugated to a C-terminal cysteine on the HER2-binding Affimers D11 and H7 via a maleimide linker and conjugation verified by mass spectrometry (Figure S2A). After 1-h treatment of HER2-positive breast cancer cell lines with 25 μg/mL (1.9 μM), AlexaFluor 488-conjugated HER2-binding Affimers showed a clear association, with both the membrane and the cytosol demonstrating internalization, whereas no association or internalization was seen in the HER2-negative MDA-MB-231 cells (Figure 2A and 2B). The internalization was significantly greater for Affimer D11 as measured by mean fluorescence intensity per cell (Figure 2C; unpaired t test; *p* = 0.02810 for AU-565 cells and *p* = 0.0410 for BT-474 cells). The internalization process was determined, in part, to be via receptor-mediated endocytosis as HER2-binding Affimers colocalized with markers of the early endosome EEA1 (Figures 2D and 2E) and, to a small degree with, the lysosome marker LAMP2 (Figures 2F and 2G). The time point used may be too early to see considerable colocalization with the latter, especially as not all Affimer appears to be internalized by this pathway. This method of internalization, via receptor-mediated endocytosis, has been seen for trastuzumab^23^ (and its derivatives^24^) as well as the targeted phototoxin DARPin-miniSOG fusion protein^25^ and is frequently accompanied by recycling of HER2 to the membrane.^24^^,^^25^ It will be interesting, in the future, to see if this HER2 recycling to the membrane occurs with the HER2-binding Affimers.

### Affimer-drug conjugates selectively reduce cell viability in HER2-expressing cell lines in a dose-dependent manner

**Figure 3:**
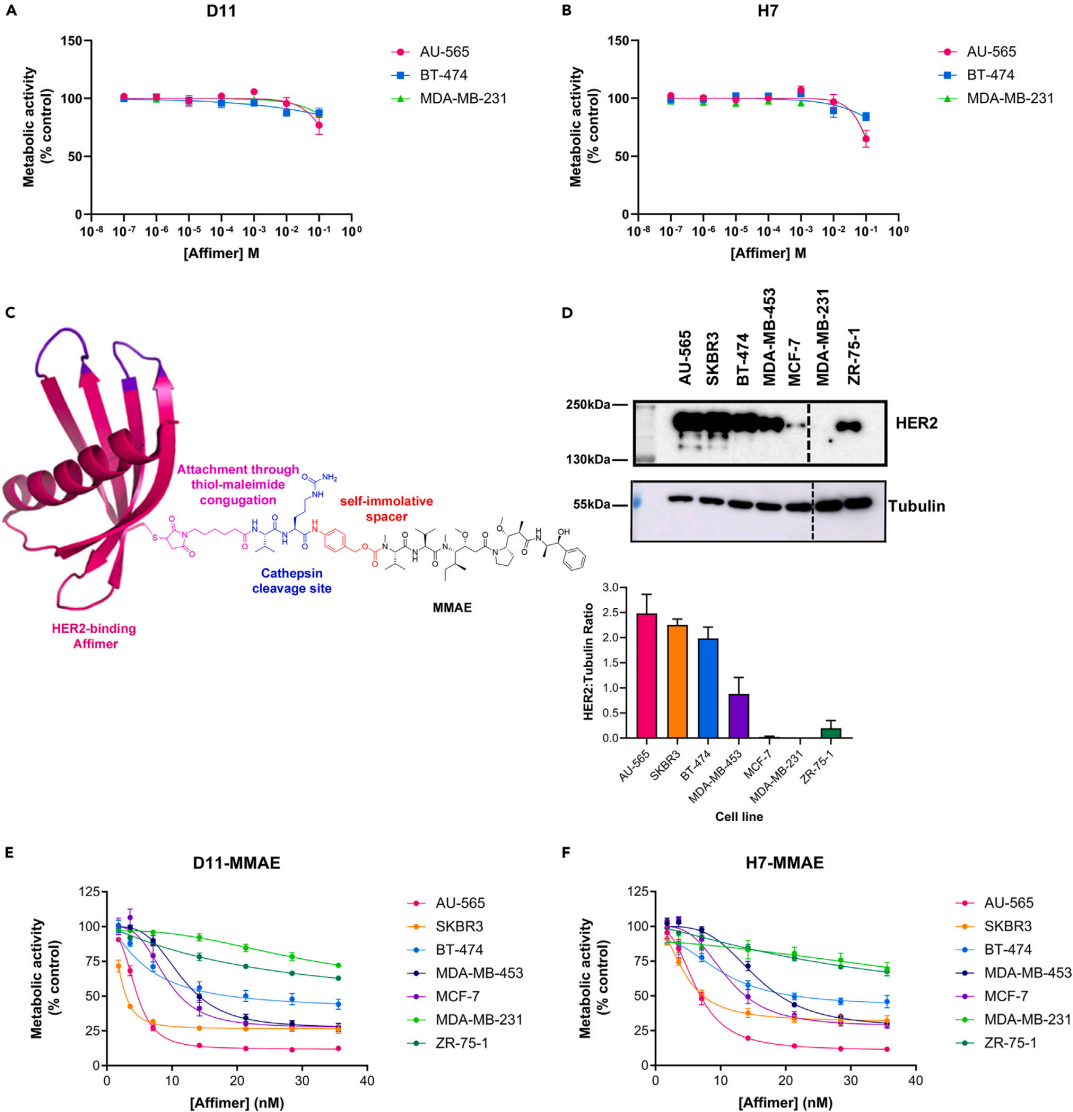
Impact of Affimers on the metabolic activity of breast cancer cell lines A range of breast cancer cell lines AU-565, SKBR3, BT-474, MDA-MB-453, MCF-7, MDA-MB-231, and ZR-75-1 were seeded one day prior to the addition of HER2-binding Affimers or HER2-binding Affimer-MMAE conjugates. Cells were incubated for a further 72 h, and metabolic activity was analyzed by AlamarBlue measurement. HER-2-binding Affimers did not affect metabolic activity in either HER2-positive cell lines AU-565 and BT474 or the HER-2-negative cell line MDA-MB-231 [(A) and (B)]. HER2-binding Affimers were conjugated with the cytotoxin MMAE via a cathepsin cleavable group and a PABC spacer (C). Seven breast cancer cell lines with varied HER2 expression level as measured by immunoblotting (D); representative blot shown; dotted line indicates removal of a lane containing lysates from a non-breast cancer cell line were treated with the Affimer-MMAE conjugates and showed dose-dependent inhibition of metabolic activity [(E) and (F)], the efficacy of which varied with HER2 expression level. All values were normalized to cells incubated with media alone. Data are mean ± SEM; dose-response curves were fitted using GraphPad Prism v 9.0, [Inhibitor] vs. response -- Variable slope (four parameters); *n* = 3 independent experiments for all panels. MMAE, monomethyl auristatin E.

Interestingly, although the HER2-binding Affimers clearly bind HER2, they did not affect cell viability directly (Figures 3A and 3B). This phenomenon has been seen with other SBPs, notably the HER2-binding Affibody (Z_HER2:342_) that alone was non-toxic toward both SKBR3 breast cancer cells and SKOV3 ovarian cancer cells,^21^ and also with monovalent HER2-binding DARPins and BT474 cells.^26^ As the Affimers alone were not cytotoxic, we used a similar approach to Sochaj-Gregorczyk et al.^13^ and conjugated the HER2-binding Affimers to monomethyl auristatin E (MMAE) via a cathepsin cleavable linker, to determine their ability to deliver the cytotoxic MMAE into cells (Figures 3C and S2B). The cytotoxic effects of the conjugates on a range of cancer cell lines with varying HER2 expression levels (Figure 3D) were determined by AlamarBlue measurement of cell metabolic activity 72 h after treatment. In HER2-expressing breast cancer cell lines, Affimer-MMAE conjugates reduced metabolic activity between 90% and 30% at the top dose of 36 nM depending on the level of HER2 expression with low nanomolar IC_50_ values (Figures 3E and 3F; Table 1). Interestingly, the Affimer-MMAE conjugates had a considerable cytotoxic effect in the MCF-7 cell line despite this cell line showing minimal HER2 expression as assessed by immunoblotting (Figure 3D). However, this result is in line with previous studies using trastuzumab-MMAE conjugates,^27^^,^^28^ which also showed that the dosing strategy is important as to whether MCF-7 cells are responsive to trastuzumab-MMAE.^28^ Thus HER2-binding Affimers can deliver cytotoxic moieties to cells in similar fashion to antibody drug conjugates and as seen previously with other SBPs, notably Affibodies where Z_HER2:2891_-DCS-MMAE showed IC_50_ values of 5.2 nM against SKBR3 cells and 24.8 nM against MDA-MB-453 cells, with IC_50_ values against the HER2 negative line MDA-MB-231 of 161.5 nM.^13^ The ability of the Affimer-MMAE conjugates to induce cytotoxicity adds support for their internalization via receptor-mediated endocytosis, as endosomes/lysosomes are a major cellular location of the cathepsin enzyme^29^ required to cleave MMAE from the Affimers to provide its cytotoxic effects.

**Table 1:** Cytotoxicity of HER2-binding Affimer-MMAE conjugates

Cell line	IC_50_ D11-MMAE (nM)	IC_50_ H7-MMAE (nM)
AU-565	5.2 ∗/÷ 1.06	7.5 ∗/÷ 1.1
SKBR3	3.7 ∗/÷ 1.2	10.6 ∗/÷1.1
BT-474	23.6 ∗/÷1.1∗∗∗∗	24.6 ∗/÷ 1.1∗∗∗∗
MDA-MB-453	17.0 ∗/÷1.1∗∗∗∗	21.7 ∗/÷ 1.0∗∗∗∗
MCF-7	14.9 ∗/÷ 1.1∗∗∗	17.5 ∗/÷ 1.1∗∗∗
ZR-75-1	61.0 ∗/÷ 1.1∗∗∗∗	68.2 ∗/÷ 1.1∗∗∗∗
MDA-MB-231	N/A	N/A

IC_50_ values calculated from Almar Blue measurements of cell metabolic activity (Figures 3E and 3F) using GraphPad Prism v 9.0, [Inhibitor] vs. response -- Variable slope (four parameters). Data are geometric mean ∗/÷ standard error of the geometric mean. ∗∗∗∗*p* < 0.0001 for both Affimer-MMAE conjugates. One way ANOVA with Dunnett’s post hoc test, compared to AU-565 cells; *n* = 3 independent experiments. MMAE, monomethyl auristatin E.

### Affimer-MMAE conjugates bind to recombinant HER2 but not HER3 with nM affinity

Next, we determined if there were any differences in the affinities of the two Affimer-MMAE conjugates for HER2 and whether or not they targeted the structurally similar HER3; to investigate these possibilities we utilized surface plasmon resonance (SPR). Neither of the Affimer-MMAE conjugates bound the ECD of HER3 (amino acids 1-643), while as expected both D11-MMAE and H7-MMAE bound the ECD of HER2 (amino acids 1-652). The K_D_ values were calculated using a 1:1 Langmuir binding model with AfD11-MMAE binding HER2 ECD with a K_D_ of 12.0 ± 0.64 nM, while AfH7-MMAE bound with a K_D_ of 76.5 ± 3.56 nM, thus AfH7-MMAE has a lower affinity than AfD11-MMAE. These affinities are in a comparable range with other HER2-targeting SBPs, as HER2-binding DARPins showed K_D_ values in the range of 7.3–28 nM when first isolated,^30^ which has subsequently been improved by error-prone polymerase chain reaction (PCR) to 91 pM,^31^ while HER2-binding Affibodies had K_D_ values of 50 nM^32^ that were improved to 22 pM.^11^^,^^12^ Thus, these data demonstrate our HER2-binding Affimers may have the scope to perform as well as other SBPs as they have not undergone any maturation processing.

### HER2-binding Affimers can be used to deliver large payloads

**Figure 4:**
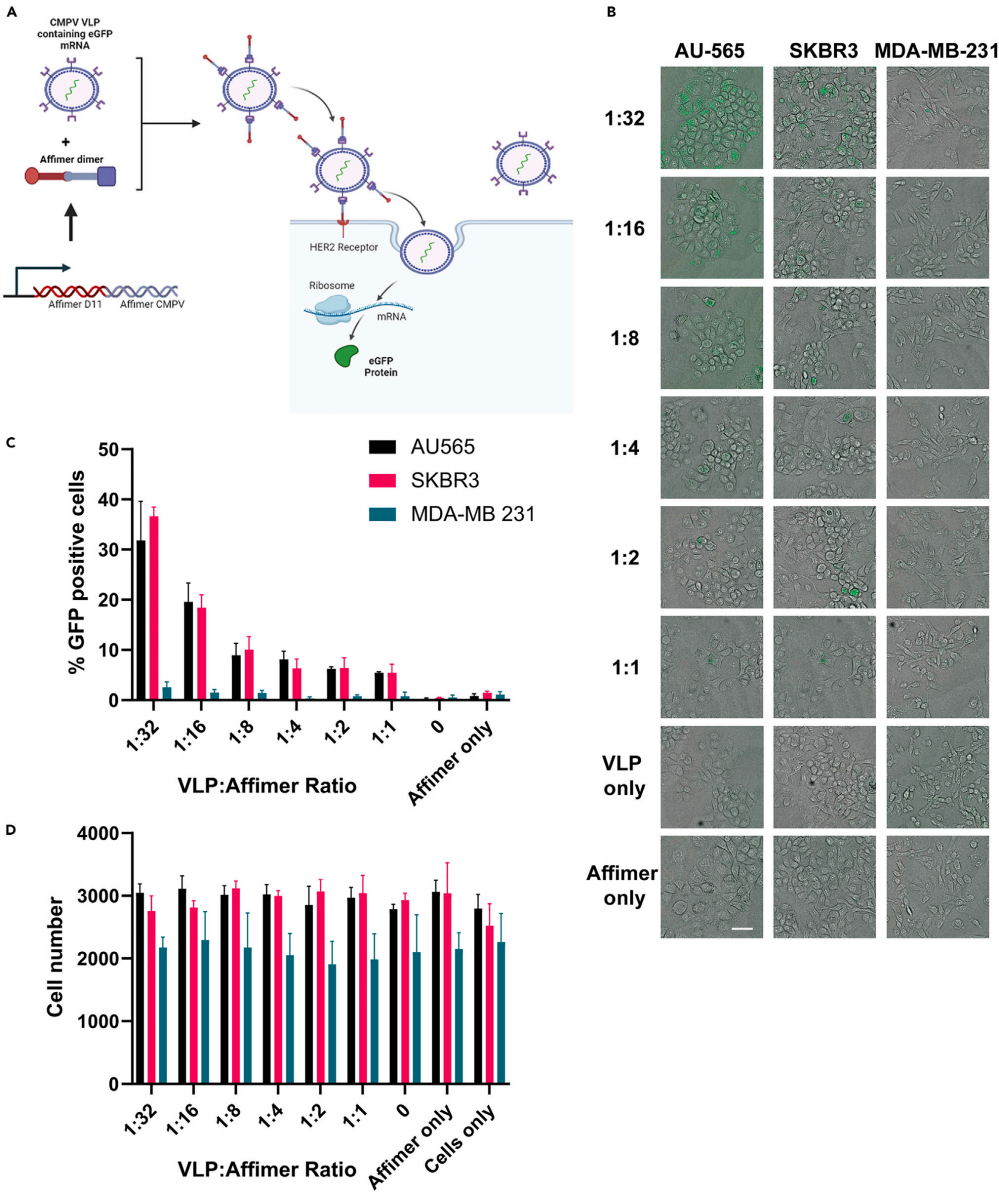
HER2-binding Affimers mediate targeted delivery of VLPs Affimer dimer containing HER2-binding Affimer D11 and CPMV-VLP-binding Affimer were used to target CPMV VLPs containing eGFP RNA to HER2-positive cells (A). Affimer dimer increased cellular uptake of eGFP RNA-containing CPMV VLPs to HER2-positive cell lines (AU-565 and SKBR3) in a dose-dependent manner (Kruskal-Wallis test; *p* = 0.0109 and *p* = 0.0099, respectively) while uptake was minimal in HER2-negative cell line MDA-MB-231 [Kruskal-Wallis test; *p* = 0.3133; (B) and (C)]. Affimer dimer:VLP conjugates were non-toxic at doses used (0.07 μM VLP and increasing ratios of Affimer dimer). Two-way ANOVA; *p* = 0.5687 (D). Data are mean ± SEM; *n* = 3 independent experiments. (A) Created with BioRender.com (2023).

Having demonstrated the ability of Affimers to deliver small cytotoxic payloads with comparable efficacy to other such systems,^12^^,^^13^^,^^20^^,^^26^^,^^30^^,^^31^^,^^32^ the assessment of their suitability to deliver larger cargos was explored. The ability to deliver larger cargos may help to overcome issues of renal clearance that occur with SBPs, as unmodified SBPs are typically cleared in a single pass as they are below the 60 kDa threshold.^33^ However, rather than select a specific molecule, we opted to use Affimers to deliver virus-like particles (VLPs), the advantage of which is the contents of the VLP may be varied; to date VLPs have been loaded with cytotoxics, small interfering RNAs (siRNAs), and proteins.^18^ Also, VLPs are known to result in an immune response that may help cancer treatments or vaccine efficacy.^34^^,^^35^^,^^36^^,^^37^ To achieve this, we used previous work demonstrating the ability of Affimers to bind to the L subunit of the coat protein of VLPs from cowpea mosaic virus (CPMV)^19^ and created an Affimer dimer consisting of the higher affinity HER2-binding Affimer—D11 and the CPMV-VLP-binding Affimer.^19^ This Affimer dimer was then incubated with VLPs produced from *Nicotiana benthamiana* leaves that were infiltrated with agrobacterial suspensions containing plasmids encoding CPMV RNA-1, VP60, and enhanced green fluorescent protein (eGFP) RNA.^38^ The composition of this VLP population was approximately 5% containing eGFP RNA, 5% containing RNA-1, and 90% empty VLPs as determined by isopycnic centrifugation over cesium chloride gradients. The integrity of the RNA-2 within the VLPs was confirmed by analysis on denaturing agarose gel electrophoresis (Figure S3). The uptake of these Affimer dimer-VLP conjugates by HER2-overexpressing and HER2-negative cell lines was analyzed by determining, by microscopy, the percentage of cells positive for eGFP expression after 72-h treatment (Figure 4A). The CPMV VLPs display 20 potential binding sites for the Affimer dimer,^19^ so we tested a variety of VLP to Affimer dimer ratios including in excess. Uptake of VLPs without Affimer dimer conjugation was minimal (0.5% or less for all cell lines tested). In HER2-overexpressing cell lines, eGFP expression increased in a dose-dependent manner as the ratio of Affimer dimer to VLPs increased (Kruskal-Wallis test *p* = 0.0109 AU-565 cells; *p* = 0.0099 SK-BR-3 cells), which was not seen in the HER2-negative, MDA-MB-231 (Kruskal-Wallis test; *p* = 0.3133) cell line when corrected for the differential levels of auto-fluorescence in each cell line (Figures 4B and 4C). None of the Affimer dimer VLP ratios reduced cell numbers, indicating they were not significantly (two-way ANOVA *p* = 0.5687) toxic to cells at the 0.07 μM concentration of VLP used in these experiments (Figure 4D).

**Figure 5:**
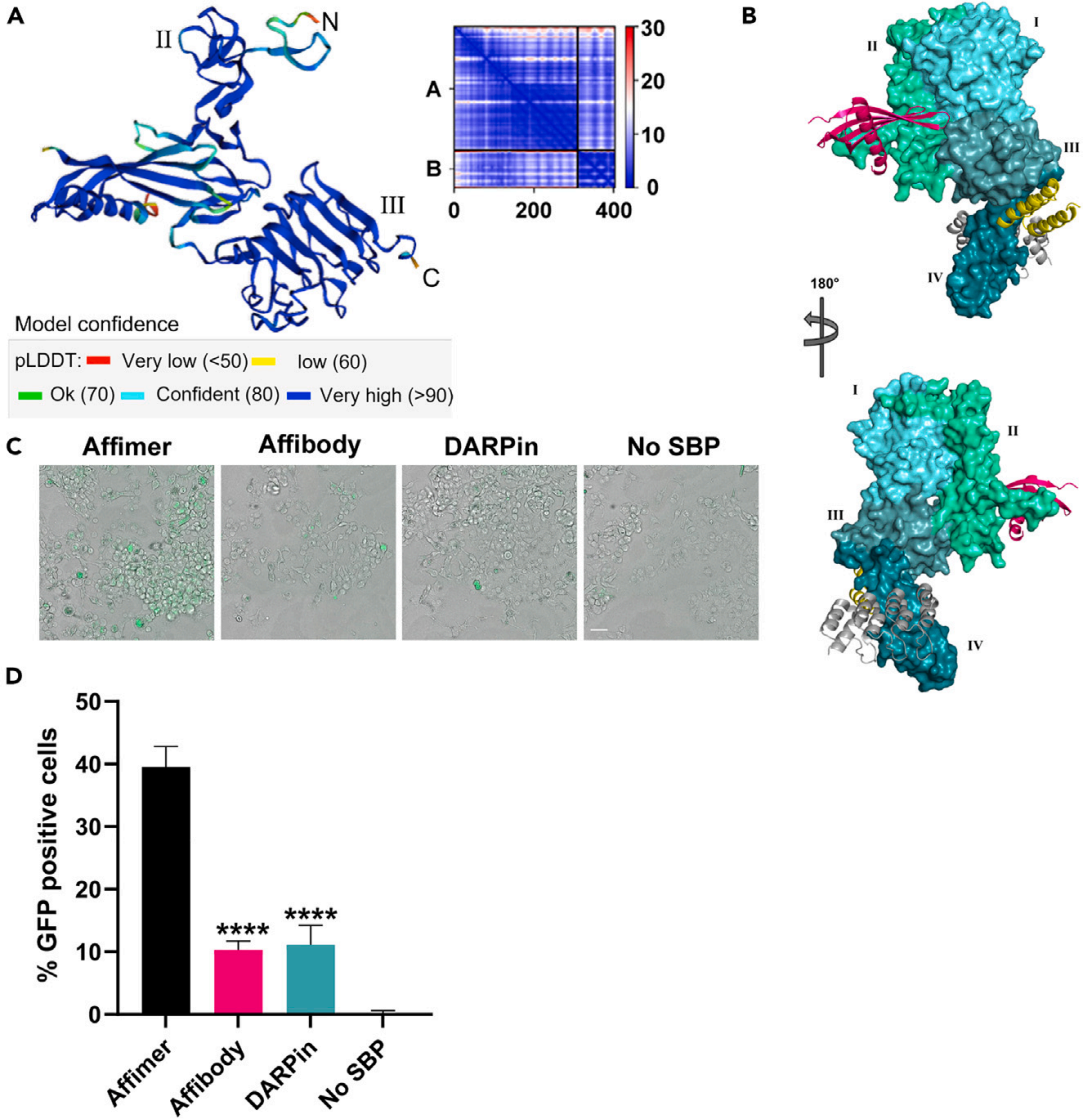
HER2-binding Affimer D11 bind different epitopes to other HER2-binding SBPs The Alphafold2-predicted model shows HER2-binding Affimer D11 level binds subdomains II and III of the HER2 ECD. The prediction is color coded based on pLDDT values that indicate the confidence level of the prediction (A) while Affibody Z_HER2:342_ (yellow; PDB code: 3MZW) binds subdomain III and DARPinG3 (gray; PDB code:4HRN) binds subdomain IV. Affimer D11 is shown in magenta (B). Replacement of HER2-binding Affimer D11 with either Affibody Z_HER2:342_ or DARPinG3 in Affimer dimer still results in an increased percentage of eGFP-positive cells over VLP alone (C) but not to the same magnitude as the Affimer dimer (D). Data are mean ± SEM; *n* = 3 independent experiments, with representative images shown in (C); scale bar is 50 μm. One-way ANOVA with Dunnett’s post hoc test ∗∗∗∗*p* < 0.0001 (D). Image created with Pymol v2.5.4 (Schrodinger LLC) (B).

Thus, we have shown that dimeric Affimers can be used for targeted delivery of VLPs to cancer cells. We then determined the versatility of this approach by replacing the HER2-binding Affimer with a HER2-binding Affibody Z_HER2:342_^12^ or a HER2-binding DARPinG3.^31^ Alphafold2 modeling^39^ shows the predicted binding site of HER2-binding Affimer D11 to be distinct from both Affibody Z_HER2:342_ and DARPinG3 binding across subdomains II and III in a similar position to pertuzumab (Figures 5A, 5B, and S4). Both the Affibody-Affimer and DARPin-Affimer SBP dimers were still capable of delivering VLPs to SKBR3 HER2-positive cells as measured by eGFP fluorescence (Figure 5C) Intriguingly, however, both the Affibody-Affimer dimer and the DARPin-Affimer dimer were less effective at delivering VLPs to HER2 positive cell lines than the dimeric Affimer (one-way ANOVA with Dunnett’s post hoc test *p* < 0.0001 for both Affibody and DARPin vs. Affimer; Figure 5D) despite both these SBPs have a higher affinity for HER2 than the HER2-binding Affimer used in the Affimer dimer.^12^^,^^31^ The decreased VLP uptake seen may result from altered biophysical properties of the Affibody and DARPin by the addition of the C terminal VLP-binding Affimer or vice versa. Further studies are required to explore this phenomenon and the precise mechanism of VLP uptake.

## Discussion

In this study, we have identified HER2-binding Affimers that are specifically internalized into HER2 expressing cells. These Affimers have nanomolar affinities similar to those seen with Affibodies and DARPins that have not undergone affinity-maturation processes.^30^^,^^32^ Such SBPs have many advantages over monoclonal antibodies in production terms, as they can be easily and reproducibly manufactured using bacterial expression systems negating the need for animals in the production process^8^^,^^9^^,^^10^ and do not require extensive post-translational modification for functionality (e.g., glycosylation).^10^ Thus, SBPs are attractive for the manufacture of therapeutics. The small size of such SBPs also increases their tissue penetration when compared to antibodies^33^^,^^40^; however, the small size of SBPs is not always an advantage, as it can lead to rapid first-pass clearance.^33^ Indeed, rapid clearance has been seen with Affimers targeting tenascin C compared with tenascin C antibodies,^8^ and this will have impacts on dosing strategy should SBP-drug conjugates get to clinic. A number of SBPs are in clinical trials and have been fused to larger proteins or conjugated with organic polymers to increase serum half-life by increasing molecule size above the 60 kDa cutoff,^33^ which may impact their tissue penetration. An additional way to overcome the limitations of the small size of SBPs, while still taking advantage of their ability to penetrate tissues more effectively than antibodies is to utilize SBPs to deliver nanoparticle cargos, as in this study using VLPs as the nanoparticles. The smaller size of SBPs mean a greater number of SBPs can bind to the nanoparticle surface^41^ and thus greater delivery capacity and tumor penetrations.^40^ However, for some applications, notably MRI, it has been shown that the optimal number of SBPs per particle for efficacious delivery is sub-maximal^42^; in other words, saturation of all particle-binding sites with SBP does not yield the most effective delivery of particles, having empty binding sites appears to improve delivery, and whether this applies to VLPs will require further investigation.

The use of SBPs and VLPs is a similar approach to that of SpyCatcher/SpyTag^43^ and DogCatcher technology.^44^ The former has been used, in a VLP context, to deliver antigens for immune activation.^45^^,^^46^ Recent work has shown that it is possible to use this approach in combination with SBPs to deliver proteins and cytotoxic drugs into cells, with Yur et al.^47^ using an epidermal growth factor receptor (EGFR)-binding DARPin as a SpyCatcher functionalization to conferred specificity for the delivery of hepatitis B VLPs containing the enzyme γCD to convert the cytotoxic pro-drug 5-FC to the cytotoxic 5-FU and GFP to MDA-MB-468 cells. This resulted in increased GFP fluorescence, but only limited cytotoxicity, which was attributed to poor cargo loading. A similar approach was used by Suffian et al.,^48^ using the HER2-binding Affibody and incorporating it into hepatitis B virus core (HBc) particles as a targeting functionalization. The Z_HER2_ ΔHBc particles showed selectivity for HER2-positive cell lines and greater accumulation in HER2-positive tumors than ΔHBc particles when administered intraperitoneally but not when administered intratumorally. Both these approaches require modification of the VLP capsid to express either the SpyCatcher protein or SpyTag peptide or the targeting SBP to permit functionalization of the VLP. In contrast, our dimeric Affimer utilizes the native L subunit found on CPMV VLPs, so functionalization is not required. Additionally, we delivered RNA rather than protein, which directly overcome the limitations associated with protein loading of VLPs that include amount of cargo, cellular localization of cargo, and release of cargo.^49^ It will be interesting to explore the loading of other cargos into the CPMV VLPs and determine their cellular effects especially as only a small percentage of the VLP populations used in this study contained eGFP RNA, the desired cargo.

CPMV VLPs may not require cargo loading when used in a cancer situation *in vivo*, as it has been reported that both wild-type VLPs and empty capsids are immunostimulatory in mice when injected intratumorally.^50^ The former had greater immunostimulatory properties and thus greater impacts on tumor growth; the authors attributed this to the presence of RNA within the wild-type VLPs that was lacking for the empty capsids. Thus, loading RNA for marker proteins such as the eGFP used in this study, prodrugs or other cargos capable of inducing cell death could enhance these effects by providing a dual approach in a single therapeutic. It will be interesting to further explore the utility and effects of dimeric Affimer-VLP conjugates in targeting tumors *in vivo*.

### Conclusions

In this study, we have isolated Affimers that bind the extracellular domain of HER2 with nanomolar affinity. We have demonstrated that these Affimers recognize and bind HER2 when present on breast cancer cells, resulting in internalization. This was then utilized to deliver the cytotoxic MMAE with clear impacts on cell viability in a HER2-dependent manner. HER2-binding Affimers are comparable to other HER2-binding SBPs, notably Affibody Z_HER2:342_^12^ and HER2-binding DARPinG3,^31^ in these attributes. The ability of all these SBPs to permit targeted delivery of nanocargos to HER2-positive cells was then demonstrated by the expression of eGFP from RNA encapsulated in CPMV VLPs by utilizing an Affimer that binds L subunit of the VLP coat protein. Thus, we have demonstrated that easily producible, low-cost SBPs can be used to specifically target VLPs to cancer cells, while further studies with a variety of VLP cargos in *in vivo* situations are needed. The work here shows potential for easily produced, low-cost targeted therapeutic alternatives to the current monoclonal antibody-based treatments for cancer.

### Limitations of the study

Although this study establishes the proof of principle that HER2-binding Affimers can specifically deliver both VLP and cytotoxic cargos into HER2-positive cells, it does not explore the mechanisms and biophysical properties of this uptake and whether this uptake occurs in primary cells/*in vivo*. An understanding of such uptake characteristics and kinetics will be needed for the HER2-binding Affimers to be exploited fully. Only *in vivo* studies, which were beyond the scope of this paper, will determine if HER2-VLP bispecific Affimer-VLP conjugates have clinical potential. Additionally, the study does not address why SBPs with higher affinities for HER2 are less effective at VLP delivery, understanding this effect will be important for determining the properties crucial for efficient targeted uptake of VLPs.

## STAR★Methods

### Key resources table

**Table:** 

REAGENT or RESOURCE	SOURCE	IDENTIFIER
**Antibodies**		

Rabbit anti-HER2	Cell Signaling Technology	Cat#2165S; RRID: AB_10692490
Rabbit anti-6xHisTag-HRP	Abcam	Cat#ab1187; RRID: AB_298652
Goat-*anti*-rabbit HRP	Abcam	Cat# ab97051; RRID: AB_10679369
Goat anti-rat HRP	Abcam	Cat# ab97057; RRID: AB_10680316
Anti-Fd-Bacteriophage-HRP	Seramun Diagnostica GmbH	Cat# A-020-1-HRP; RRID:N/A
Mouse anti-EEA1	BD Bioscience	Cat#610456; RRID: AB_397829
Rabbit anti-LAMP2	GeneTex	Cat# GTX103214; RRID: AB_10615814
AlexaFluor™ 594 goat anti-rabbit	Invitrogen	Cat#A11012; RRID: AB_2534079
AlexaFluor™ 594 goat anti-mouse	Invitrogen	Cat#A11005; RRID: AB_2534073

**Bacterial and virus strains**		

BL21 Star™ (DE3)	Invitrogen	Cat# C601003
XL1-Blue Super-competent	Aglient	Cat#200249
ER2738	Lucigen	Cat#60522-1
Rhizobium radiobacter (agrobacterium)	Firefly	Cat# 01198P

**Chemicals, peptides, and recombinant proteins**		

HER-2 ECD-FC tagged	Sino Biologicals	Cat#10004-H04H
HER3 ECD protein	Sino Biologicals	Cat# 10201-H08H
AlexaFluor 488™ C 5 maleimide	Invitrogen	Cat# A10254
Auristatin E (MMAE) maleimide with a cathepsin-cleavable linker and *p*-aminobenzyloxycarbonyl spacer	Broadpharm Therapeutics	Cat# BP-23969

**Critical commercial assays**		

AlmarBLue	ThermoFisher Scientific	Cat#DAL1025

**Experimental models: Cell lines**		

AU-565	ATCC	Cat# CRL-2351; RRID:CVCL_1074
SKBR3	ATCC	Cat# HTB-30; RRID:CVCL_0033
BT-474	ATCC	Cat# HTB-20; RRID:CVCL_0179
MDA-MB-453	ATCC	Cat# HTB-131; RRID:CVCL_0418
MCF-7	ATCC	Cat# HTB-22; RRID:CVCL_0031
MDA-MB-231	ATCC	Cat# HTB-26; RRID:CVCL_0062
ZR-75-1	ATCC	Cat# CRL-1500; RRID:CVCL_0588

**Experimental models: Organisms/strains**		

*Nicotiana benthamiana*	John Innes Center	N/A

**Recombinant DNA**		

CPMV RNA-1 and and	John Innes Center	N/A
VP60 (pEAQ-RNA1-Int)	John Innes Center	N/A
pHREAC-VP60	John Innes Center	N/A
Affimer phage pBSTG	This study	N/A
AffimerC HisTag pET11	This Study	N/A

**Software and algorithms**		

CellReporterXpress v2.8.2	Molecular Devices	https://www.moleculardevices.com/products/cellular-imaging-systems/acquisition-and-analysis-software/cellreporterxpress
GraphPad Prism v9.02	GraphPad Software	https://www.graphpad.com/features
ImageJ	ImageJ software	https://imagej.net/ij/

### Resource availability

#### Lead contact

Further information and requests for resources and reagents should be directed to and will be fulfilled by the lead contact, Darren Tomlinson (d.c.tomlinson@leeds.ac.uk).

#### Materials availability

The Affimer constructs will be made available under a University standard MTA. VLPs can be bought directly from Leaf Expression Systems or via collaboration with GL.

#### Data and code availability

• All data reported in this paper will be shared by the lead contact upon request
• This paper does not report original code.
• Any additional information required to reanalyze the data reported in this paper is available from the lead contact upon request.

### Experimental model and study participant details

#### Cell culture

AU-565 (RRID:CVCL_1074), SKBR3 (RRID:CVCL_0033), BT-474 (RRID:CVCL_0179), MDA-MB-453 (RRID:CVCL_0418), MCF-7 (RRID:CVCL_0031), MDA-MB-231 (RRID:CVCL_0062) and ZR-75-1 (RRID:CVCL_0588) were purchased from ATCC. AU-565, BT-474, MCF-7, MDA-MB-453 and ZR-75-1 cell lines were maintained in RPMI-1640 (Gibco, Paisley, UK) supplemented with 10% fetal bovine serum (FBS; Gibco), whilst SKBR3 and MDA-MB-231 cell lines were maintained in Dulbecco’s Modified Eagle’s Medium (Gibco) supplemented with 10% FBS, at 37°C and 5% CO2. All cell lines used in this study were mycoplasma free and their identities were confirmed by STR profiling.

#### Plant cultivation

*N. benthamiana* (RRID:NCBITaxon_4100) seeds were collected from plants cultivated in-house from a stock that was obtained from the John Innes Center (Norwich, UK). The seeds were sown on damp Levington Advance Seed Modular F2 Professional Growing Media (Berrycroft Stores Ltd, Cambridge, UK) in HSP full seed trays; 34.4 × 21.4 × 5.2 cm, with propagator lids (Fargro, West Sussex, UK). The seeds were germinated in a Weiss Technik SGC 120 controlled environment cabinet at 22°C, under mixed G2 pink (35% light intensity) and NS12 white (35% light intensity) LED lighting operating a 16 h light/8 h dark cycle with 70% humidity, and daily watering. After two weeks the seedlings were potted out into Teku MQD 9 × 9 × 9.5 cm pots (Fargro, West Sussex, UK) containing the same compost used for seedling germination and transferred to a Weiss Technik SGR223 (LED) controlled environment room (CER) where they were cultivated under identical conditions as above for a further three weeks prior to infiltration. Following infiltration, the plants were returned to the CER for a further six days before the leaves were manually harvested and used for purification.

### Method details

#### Isolation of HER2-binding affimers

Target biotinylation, selection of Affimers by phage display and phage ELISA against the ECD of HER2 was as previously described.^8^^,^^9^ Briefly, biotinylated HER2 ECD His-tagged (10004-H02H, Sino Biologicals, Eschborn, Germany; EZ-Link NHS-Biotin, Thermo Scientific, Waltham, MA; 5-fold molar excess) was immobilised on blocked (2x blocking buffer, Sigma) streptavidin wells. The Affimer phage library was applied for 2 h and unbound phage removed by PBS-T washes (27 times). Bound phage were eluted in a two-phase step, firstly with 0.2 M Glycine pH 2.2 neutralised with 15 mL of 1 M Tris-HCl, pH 9.1 and then 7.18 M Triethylamine, pH 11 neutralised with 1 M Tris-HCl, pH 7. Three panning rounds were undertaken and after the final panning round 32 randomly picked colonies were used in phage ELISA with positive clones sent for sequencing.^7^^,^^8^^,^^9^ Two additional panning rounds with HER2 ECD applied with the phage were undertaken to identify Affimers that bound in the presence of HER2.

Three additional panning rounds were performed on a 6-well plate contained a monolayer of fixed-cells using the phage from pan 3 above. Approximately 1.2x10^6^ of MCF-7 (non-target cells) and MDA-MB-453 (target cells) cells were fixed with 4% paraformaldehyde (SigmaAldrich, Gillingham, UK) for 15 min at room temperature. Prior to overnight blocking with 2X casein buffer at 4°C, fixed cells were washed three times with PBS solution supplemented with 0.1% sodium Azide. Following overnight blocking, 100 mL of eluted phage from the third panning round (1:10 dilution prepared with 10X casein blocking solution) were pre-panned on a blocked well containing no cells followed by another 40 min on fixed MCF-7 cells at room temperature. Then, an equal volume of pre-panned phage were added on target and non-target fixed-cells and incubated for 2 h at 50 rpm shaking speed. Prior to elution, wells were washed 10 times (5 min each) with 1 mL of PBS, pH 7.4. Cell-bound phage were then eluted as detailed above, after the final panning round 48 randomly picked colonies were used in phage ELISA^7^^,^^8^^,^^9^ against approximately 8 ×10^3^ fixed MCF-7 or MDA-MB-453 cells in 96 well plates with positive clones sent for sequencing identifying 2 unique sequences.

#### Production of HER2-binding affimers

The eight unique sequences were cloned into pET11a, with or without a C terminal cysteine, using the NheI and NotI sites. The SBP dimer coding sequences were designed with a GSGGSGGSGG linker sequence separating the HER2 -binding SBP sequences from the VLP binding Affimer sequence, and differed in codon usage for each Affimer scaffold sequence. The sequences were synthesised by GenScript Biotech (Piscataway, NJ) and inserted between the NdeI/BamHI sites of pET-11a. HER2-binding Affimers and SBP dimers were produced in BL21 STAR (DE3) E. coli (C601003, Life Technologies, Invitrogen) and affinity purified using Ni-NTA resin as previously described.^7^^,^^8^^,^^9^

#### Protein extraction, affinity precipitation and immunoblotting

Mammalian cell lines were expanded to 80% confluency and pelleted by centrifugation at 1000 × *g* for 5 min. Pellets were lysed in with 1 mL mammalian cell lysis buffer (25 mM Tris-HCl, 150 mM NaCl, 5% glycerol (v/v), 1% NP-40 (v/v), pH 7.0) supplemented with Pierce Halt EDTA free protease inhibitor cocktail (ThermoFisher) and incubated at 4°C with rotation for 35 min, followed by centrifugation at 12,000 × *g* for 20 min. Protein concentration was determined using a Pierce BCA Protein Assay Kit (ThermoFisher) as per manufacturer’s instructions.

For affinity precipitation 2 mg/mL mammalian lysates were mixed with 0.65 mg/mL Affimers in mammalian lysis buffer and incubated overnight at 4°C with rotation. Affimer-HER2 complexes were isolated using Ni-NTA chromatography. Briefly, the overnight mixtures of Affimer and cell lysate were incubated with Ni-NTA slurry (Abcam, Cambridge, UK) for 90 min at 4°C with rotation. Saturated slurry was then washed 5 times (50 mM NaH_2_PO_4_, 500 mM NaCl, 20 mM Imidazole, 0.1% Tween 20 (v/v) pH 7.4) and eluted in 50 μL elution buffer (50 mM NaH_2_PO_4_, 500 mM NaCl, 300 mM Imidazole, 20% glycerol, pH 7.4) at 4°C. Samples were mixed with 4 × loading buffer (200 mM Tris-HCl, pH 6.8, 20% (v/v) glycerol, 8% (w/v) SDS, 0.4% (w/v) bromophenol blue, 20% (v/v) β-mercaptoethanol (Fisher Scientific, Loughborough, UK) and heat denatured at 95°C for 5 min and run on 8 or 15% SDS-PAGE gels as detailed below.

For immunoblotting 10–15 μg of lysates were heated with 4× loading buffer for 5 min at 95°C, loaded onto 8 or 15% SDS-PAGE gels and run at 150 V before transfer to nitrocellulose membrane using the BioRad Transblot Turbo (BioRad, Hercules, CA). Membranes were blocked in 5% (w/v) milk (SigmaAldrich) in TBS-T (0.1% v/v) for 1 h room temperature and incubated with primary antibody rabbit anti-HER2 (2165S, Cell Signaling Technology, Danvers, MA; 1:10,000; overnight at 4°C), rabbit anti-6xHisTag HRP (ab1187, Abcam; 1:10,000; 1 h at room temperature) or rat anti-α-tubulin (MCA78G, BioRad; 1:3000), in 5% (w/v) milk. Membranes were washed 3 times in TBS-T and incubated with secondary antibody goat anti-rabbit HRP (ab97051, Abcam; 1:10,000) or goat anti-rat HRP (ab97057, Abcam; 1:10,000) in 5% (w/v) milk for 1 h at room temperature if required. Membranes were washed 3 times in TBS-T and developed with Immobilon Forte Western HRP Substrate (Millipore, Burlington, MA). Membranes were imaged on an Amersham Imager 600 (GE Healthcare, Chicago, IL) and images analyzed with ImageQuant TL v8.1.0.0 (GE Healthcare).

#### Conjugation of affimers

Affimers were conjugated to AlexaFluor 488 TM C 5 maleimide (A10254, ThermoFisher), or monomethyl auristatin E (MMAE) maleimide with a cathepsin-cleavable linker and *p*-aminobenzyloxycarbonyl spacer (BP-23969, Broadpharm Therapeutics, San Diego, CA) via a cysteine residue in the C-terminal domain. Purified Affimers were diluted to 0.5 mg/mL in PBS and incubated with washed immobilised TCEP disulphide-reducing gel (ThermoFisher) for 1 h at room temperature. Affimers were then incubated with AlexaFluor 488 or MMAE respectively for a further 2 h at room temperature. Affimer-MMAE conjugates were quenched with β-mercaptoethanol (FisherScientific). All conjugates were desalted using 7 MWCO 0.5 mL Zeba spin columns (ThermoFisher) as per manufacturer’s instructions and stored at 4°C. Conjugation was confirmed by mass spectrometry. Protein desalting and mass analysis was performed by liquid chromatography mass spectrometry (LC-MS) using an M-class ACQUITY UPLC (Waters UK, Manchester, UK) interfaced to a Xevo QToF G2-XS mass spectrometer (Waters UK, Manchester). Samples were diluted to 5 μM using 0.1% TFA. 1 μL of the 5 μM sample was loaded onto a MassPREP protein desalting column (Waters UK, Manchester) washed with 10% solvent B in A for 5 min at 25 μL min^−1^. After valve switching, the bound protein was eluted by a gradient of 2–40%

#### Immunofluorescence using confocal microscopy

Cells were seeded onto coverslips in a 24 well plate at 3 × 10^4^ cells/mL. The following day coverslips were washed once in 1 × DPBS and incubated with 25 μg/mL Affimer-AlexaFluor 488 conjugate in OptiMEM (Gibco) for 1 h. Cells were washed 3 × in PBS prior to fixing in 4% paraformaldehyde for 15 min followed by permabilisation with 0.1% Triton X-100 in PBS for 5 min. Cells were washed in PBS and blocked with 1% milk (Sigma Aldrich) overnight at 4°C. Cells were incubated with primary antibodies rabbit anti-HER2 (2156S, Cell Signaling Technology; 1:2000), mouse anti-EEA1 (610456, BD Bioscience, Wokingham, UK; 1:200) or rabbit anti-LAMP2 (GTX103214, GeneTex, Irvine, CA; 1:200) in 1% milk for 1 h at room temperature followed by 3 × PBS washes and 1 h incubation with AlexaFluor 594 goat anti-rabbit (A11012, Invitrogen; 1:1000) or AlexaFluor 594 goat anti-mouse (A11005, Invitrogen; 1:1000) in 1% milk for 1 h at room temperature. Cells were washes 3 × PBS and mounted using ProLong Gold antifade reagent and DAPI (Invitrogen, Cat. No. P36935). Cells were imaged using a Zeiss LSM880 confocal microscope. Affimer intensity per cell was measured using ImageJ version 1.54.

#### Surface plasmon resonance

Affimer-MMAE conjugate affinities for HER2 and HER3 ECD were determined by surface plasmon resonance (SPR) using a BIAcore T200 (GE Healthcare Europe GmbH). Recombinant HER2 and HER3 ECD protein (10004-H04H and 10201-H08H, Sino Biologicals) immobilised by amine coupling onto CM5 chip (BIAcore). Biacore experiments were performed at 25°C in PBS pH 7.4 supplemented with 0.1% Tween 20 (v/v). Affimer-MMAE conjugates were injected at 0.78, 1.56, 3.13, 6.25, 12.5, 25, 50, 100 and 200 nM for 3 min at a flow rate of 30 μL min^−1^, followed by a buffer wash for 10 min to follow complex dissociation. The on- and off-rates and KD parameters were obtained from a global fit to the SPR curves using a 1:1 Langmuir model, using the BIAevaluation software v 3.1 (GE Healthcare). Quoted KD values are the mean ± SEM of three replicate measurements.

#### Cell viability

Cells were seeded in a 96 well plate on day 0 for an intended 40% coverage of the plate on day 1. Affimer-MMAE conjugates were added at the indicated concentrations. Cells were incubated for a further 72 h. Media was removed and prewarmed media containing 10% AlamarBlue (ThermoFisherScientific) was added and incubated on cells at 37°C, 5% CO2 with humidity for approximately 2–4 h until a color change was visible. Fluorescence was measured on a Tecan Spark microplate reader (Tecan Trading AG) with an excitation wavelength of 570 nm and emission wavelength of 590 nm on a Tecan Spark microplate reader (Tecan Trading AG).

#### VLP production

CPMV- like particles containing RNA encoding eGFP were produced using a modified purification procedure, adapted from the protocols described by Peyret et al.^38^^,^^51^ Briefly, *N. benthamiana* leaves were vacuum infiltrated with a mixture of agrobacterial suspensions containing plasmids encoding CPMV RNA-1 and VP60 (pEAQ-RNA1-Int and pHREAC-VP60, respectively) together with plasmid (pEAQ-eGFP), a slightly modified version of pEAQ-GFP encoding eGFP in place of wild-type GFP. Prior to infiltration, the bacterial strains were cultured overnight in 2 L Erlenmeyer flasks containing LB media with kanamycin (50 μg/mL) and carbenicillin (100 μg/mL) selection. At the point of infiltration, the three cultures were each diluted to a final OD600 of 0.4 and combined in the presence of 0.1 mM acetosyringone. Six days after infiltration, the leaf tissue was harvested and mechanically homogenised using a 4L preparative blender (Waring, Connecticut, USA) in TBS pH7.0 containing 10 mM sodium metabisulfite. The homogenate was clarified by filtration using a 1 μm Polypropylene bag filter (Fileder Filter Systems Ltd, UK), followed by centrifugation at 11,000 × g for 15 min. The supernatant was removed and further clarified by depth filtration using a 0.45/0.65 μm Whatman Polycap filter (Cytiva, UK) followed by ultrafiltration in retentate mode using a 100 kDa PES filter (Repligen, Waltham, USA). The clarified plant extract was passed through a 0.45 μm PES bottle top filter (Thermo Fisher Scientific) before being treated with ice-cold 5% PEG6000 and 0.25 M NaCl for 1 h to precipitate the RNA-containing VLPs. Following centrifugation at 11,000 × g for 1 h, the supernatant was removed, and the pellets resuspended in 20 mM Sodium Phosphate pH7.0. The purified VLPs were further clarified by centrifugation at 20,000 × g for 1 h prior to filter sterilisation using a 0.22 μm PES filter and storage at +4°C. The VLP distribution was assessed using 12 mL self-forming 41% (w/v) CsCl gradients buffered with 10 mM sodium phosphate pH 7.0, centrifuged at 40,000 rpm in a TH641 rotor at 15°C for 24 h. The gradients were fractionated using a BioComp fractionator equipped with a Triax flow-cell to monitor A280. RNA extracted from the fractionated VLPs was analyzed by electrophoresis through formaldehyde-containing 1.3% (w/v) agarose and visualised by ethidium bromide staining.

#### VLP uptake assay

AU-565, SKBR3 or MDA-MB-231 cells were plated at 5 × 10^4^ cell/mL in 96 well plates (Viewpoint, PerkinElmer, Waltham, MA). Twenty-four hours later VLPs were diluted to a concentration of 0.7 μM in DPBS and mixed at a variety of ratios with HER2-binding Affimer D11-VLP binding Affimer dimer in DPBS and incubated at room temperature for 10 min with agitation. Affimer-dimer VLP mixtures were then added to AU-565, SKBR3 or MDA-MB-231 cells at a 1:10 dilution and incubated for 72 h. Cells were then rinsed with DPBS before fixation in 4% paraformaldehyde, washed 3 times with PBS and imaged on ImageXpress Pico (Molecular Devices) and analyzed in CellReporterXpress v.2.8.2 (Molecular Devices) for both eGFP uptake and cell numbers. For Affibody- and DARPin-Affimer dimers, SKBR3 cells were plated as detailed above and SBP-dimers incubated with VLPs at a 1:32 ratio only and treated as detailed for Affimer-dimers above.

#### Alphafold2 structure prediction

The sequences of truncated HER2 (DII/DIII) and Affimer D11 were submitted to AlphaFold2 w/MMseq2 (ColabFold v1.5.2) (https://colab.research.google.com/github/sokrypton/ColabFold/blob/main/

AlphaFold2.ipnyb). The DII/DIII:Affimer D11 complex was modeled using a pipeline adapted to run 24 recycles with a recycle early stop tolerance of 0.0. The model superimposed on experimentally determined structures of HER2 to evaluate the accuracy of model.

### Quantification and statistical analysis

Statistical analyses (Unpaired T-test, One-way ANOVA, Kruskal Wallis and two-way ANOVA) detailed in the manuscript text and figures were carried out in GraphPad Prism 9.00 software (GraphPad Software, La Jolla, CA). ∗ = *p* < 0.05, ∗∗∗∗ = *p* < 0.0001. Statistical assumptions of equal variance for one-way ANOVA were tested with Brown-Forsythe test and normality was tested with Shapiro-Wilk tests. Dose-response curves were fitted using GraphPad Prism v 9.0, [Inhibitor] vs. response -- Variable slope (four parameters). The data plotted and the number of experimental repeats is detailed in the figure legends.
