# Supplementary material for: Affimer reagents enable targeted delivery of therapeutic agents and RNA via virus-like particles
Source: iScience. 2024 Jul 5;27(8):110461. doi: 10.1016/j.isci.2024.110461 (PMC11298639; doi:10.1016/j.isci.2024.110461)

iScience, Volume *27*

Supplemental information

Affimer reagents enable targeted delivery of therapeutic agents and RNA via virus-like particles

Sophie E. Roberts, Heather L. Martin, Danah Al-Qallaf, Anna A. Tang, Christian Tiede, Thembaninkosi G. Gaule, Albor Dobon-Alonso, Ross Overman, Sachin Shah, Hadrien Peyret, Keith Saunders, Robin Bon, Iain W. Manfield, Sandra M. Bell, George P. Lomonossoff, Valerie Speirs, and Darren C. Tomlinson

SUPPLEMENTARY FIGURES

Supplementary Figure 1. Relative HER2 expression in 62 breast cancer cell line using data mined from the Human Protein Atlas (https://www.proteinatlas.org/). Related to Figure 1. Cell lines reported in this study are indicated by asterisks.

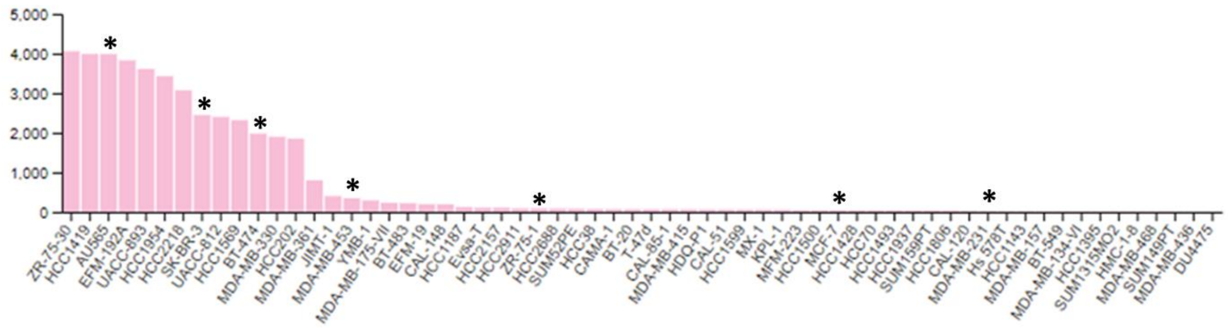

Supplementary Figure 2. Accurate mass spectra for conjugated HER2-binidng D11 and H7 Affimers. Related to Figures 2 and 3, and Table 1. Affimers conjugated to AlexaFluor488 (**a)**) show a main *m/z* peak corresponding to the predicted mass of the D11- (upper panel) and H7- (lower panel) AlexaFluor488 conjugates, indicated as peak A in both spectra. Affimers conjugated to MC-VC-PBAC-MMAE (**b)**) shows a major *m/z* peak for D11- (upper panel) and H7- (lower panel) MMAE conjugates (minus methionine), indicated as peak B in both spectra, the secondary *m/z* peak (indicated as peak A) is an unidentified secondary product. All traces show a lack of free Affimer *m/z*, indicating that conjugation occurred to completion.

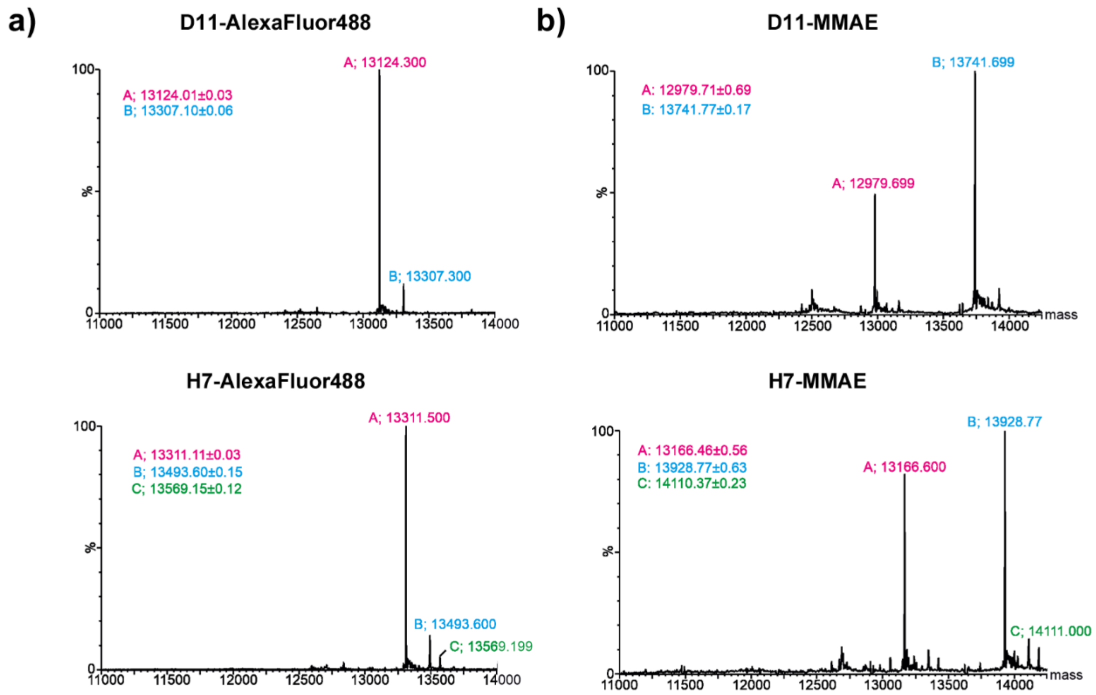

Supplementary Figure 3. Composition of the VLP sample used. Related to Figure 4 and 5. Fractionation of VLP sample used in this study over 41% (w/v) CsCl gradient (**a)**). RNA was extracted from the pooled fractions and run on formaldehyde-containing 1.3% (w/v) agarose. Lane 2 represents a control sample showing the positions of GFP RNA (1.4kb) and CPMV RNA-1 (6kb) and Lane 3 shows the 19-20 fractions contained eGFP (**b)**).

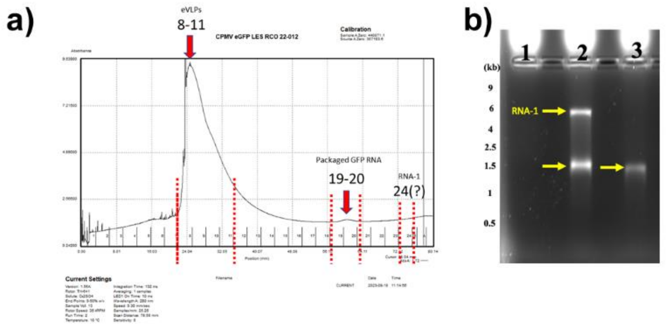

Supplementary Figure 4.

Alphafold2 modelled HER2 ECD binding site of Affimer D11 (magenta) compared with pertuzumab (Khaki; PDB Code: 1S78). Related to Figure 5. Image created with Pymol v2.5.4 (Schrodinger LLC).

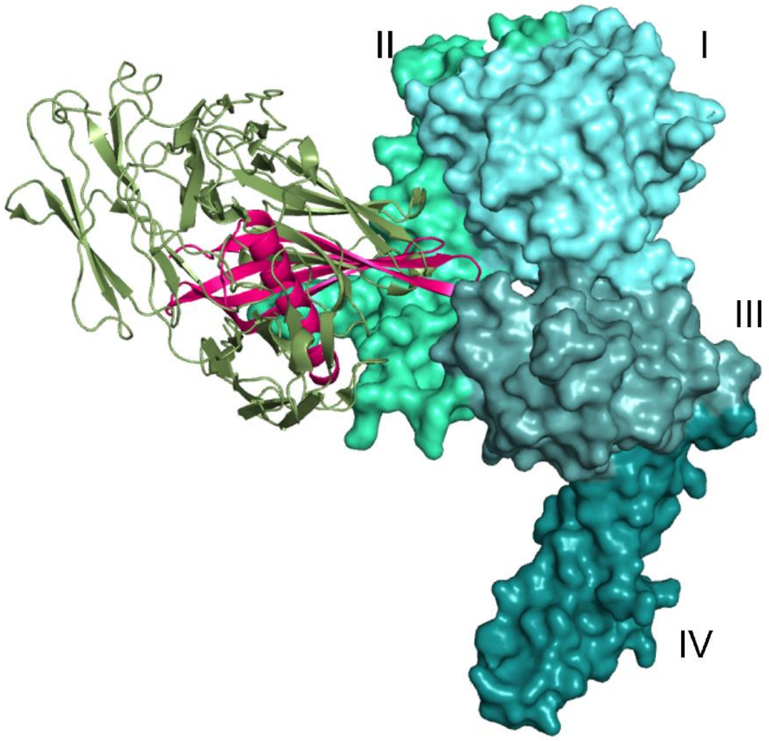

Supplement: Document S1. Figure S1–S4 [file mmc1.pdf]
